# Profiling the Prognosis of Gastric Cancer Patients: Is It Worth Correlating the Survival with the Clinical/Pathological and Molecular Features of Gastric Cancers?

**DOI:** 10.1155/2013/196541

**Published:** 2013-12-24

**Authors:** Laura Lorenzon, Paolo Mercantini, Mario Ferri, Marco La Torre, Alessandra Sparagna, Genoveffa Balducci, Giulia Tarantino, Adriana Romiti, Emanuela Pilozzi, Vincenzo Ziparo

**Affiliations:** ^1^Surgical and Medical Department of Translational Medicine, Sant'Andrea Hospital, Faculty of Medicine and Psychology, University of Rome “La Sapienza”, Via di Grottarossa 1035-39, 00189 Rome, Italy; ^2^Department of Clinical and Molecular Medicine, Sant'Andrea Hospital, Faculty of Medicine and Psychology, University of Rome “La Sapienza”, Via di Grottarossa 1035-39, 00189 Rome, Italy

## Abstract

*Background*. The prognosis of gastric cancer patients still remains poor. The aim of this study was investigating the prognostic value of several clinical/pathological/molecular features in a consecutive series of gastric cancers. *Methods*. 150 R0 gastrectomies plus 77 gastric cancer patients evaluated for the HER2 overexpression were selected. Survival was calculated and patients stratified according to the stage, the T-stage, the LNRs, the LNH, and the HER2 scoring system. ROC curves were calculated in order to compare the performance of the LRN and LNH systems. *Results*. Prognosis correlated with the stage and with the T-stage. We documented a statistical correlation between the LNRs and the survival. Conversely, a LNH > 15 did not correlate with the outcomes. The ROC curves documented a significant performance of the LRN system, whereas a statistical correlation was documented for the LNH exclusively with the endpoint of disease-free survival. We documented a trend of worse prognosis for patients with an HER2 overexpression, even though it was not of statistical value. *Conclusion*. The LNR and the evaluation of the HER2 overexpression might be useful since they correlate with survival, might identify patients with a higher risk of recurrence, and might select patients for a tailored medical treatment.

## 1. Introduction

Gastric cancer is a global health issue and it has been recognized as the 4th most common cancer worldwide [1]; nevertheless, nowadays the prognosis of gastric cancer patients still remains poor. In Italy, it has been recently ascribed as the third most relevant cause of death related to cancer among males and the fifth among females [2].

Because of the health and social importance of this disease, several lines of research have been followed in order to identify significant prognostic factors of cancer-related survival and mortality, first of all in the field of the nodal (N) staging systems. In the past, the N stage has been defined by the location of the positive nodes in relation to the gastric cancer, since involved nodes within 3 cm were staged as N1 and the metastatic nodes positioned more than 3 cm from the primary tumor were regarded as N2 tumors [3]. In 1997 the AJCC staging manual based the N stage on the number of metastatic nodes [4]: cancers were recognized as N1 if displaying 1–6 positive nodes, N2 if exhibiting 7–15 positive nodes, and N3 if reporting more than 15 involved nodes [4]. In addition, in order to improve the accuracy of the nodal staging, it was recommended that at least 15 lymph nodes should be examined in order to define the N0 stage, highlighting the prognostic assessment of the lymph-node harvest (LNH) [5].

In the current edition of the staging manual, gastric cancer nodal staging has been additionally changed and patients ascribed in the N1 group if displaying 1-2 nodes involved, N2 if presenting with 3–6 positive nodes, N3a if the pathologic examination has been consistent with 7–15 nodal metastasis, whereas N3b if it documented more than 16 positive nodes [6].

In 1997 a Japanese study proposed the ratio between metastatic and examined lymph-nodes (lymph-node ratio, LNR) as a new, original, and significant prognostic factor for Stage IV stomach cancers [7].

Moreover, in the recent literature, few studies suggested that the LRN is a more effective prognostic factor of survival comparing with the number of metastatic nodes [8–10] and thus it might improve the current staging system.

Interestingly, the overexpression of the human epidermal growth factor receptor 2 (HER2), member of the type I receptor tyrosine kinase (RTK) family, has been reported in gastric adenocarcinoma as an independent unfavourable prognostic factor [11, 12].

Furthermore a humanized monoclonal antibody against HER2 was found to be promising in therapy of patients with gastric cancer [13].

On the basis of this background we aimed this study to investigate the prognostic value of several clinical/pathological and molecular features in relationship with the survivals of a consecutive series of patients who have undergone curative gastric resection (R0) for adenocarcinoma of the stomach and/or evaluated for the HER2 overexpression.

Specifically we aimed this study to the prognostic assessment of the Stage, T-Stage, the LRN, the LNH and the HER2 immuno-histo-chemistry (IHC) scoring systems.

## 2. Materials and Methods

### 2.1. Patients and Setting

All the surgical, clinical, and pathological data of the consecutive patients who have undergone resection for gastric/cardial carcinomas at the Surgery 1 Unit of the Sant'Andrea Hospital, Faculty of Medicine and Psychology, “La Sapienza” University of Rome, and/or evaluated for the HER2 overexpression from March 2003 to December 2011, were recorded in a prospective database by the authors of the present study (*n* = 227 gastrectomies) and were retrospectively reviewed. Authorization of the ethical board was not required for this retrospective investigation, but signed consent for the treatment and the evaluation of data was obtained from all patients before the procedures.

### 2.2. Selection Criteria

Patients were selected if undergoing a R0 resection for an adenocarcinoma of the stomach or cardia (Siewert's class III). 13 patients were excluded due to a histologic diagnosis other than adenocarcinoma and 18 patients due to R1-R2 resections. 46 patients were lost to follow-up, leaving for the analysis 150 patients. This group of patients has been selected for the correlation between stage, T-Stage, LNR, and LNH with survival. Furthermore, for the purpose of this investigation, we included in this study 77 gastric cancer patients who have undergone IHC evaluation of the HER2 overexpression (in the surgical specimen or in the cancer biopsy) enrolled at our Department and at the Department of Oncology of the Sant'Andrea Hospital, Faculty of Medicine and Psychology, “La Sapienza” University of Rome in the same mean time.

### 2.3. Records

All data were reviewed, including tumor location and surgical procedure, patient's clinical and demographics data (age at the time of surgery, sex), and possible adjuvant treatments.

Surgical procedures standardized by the team included a gastrectomy (according with the tumor's localization) plus a lymphadenectomy consistent with D1 nodal dissection plus resection of the nodes of the celiac trunk and of the hepatic pedicle.

The pathological records included the macro/microscopic description of the tumor and of the surgical specimen, plus the TN category along with the Stage of the disease. The pathological data also reported the LNH and number of metastatic lymph nodes and tumor's grading.

### 2.4. LNR and LNH

The LNR has been calculated as the ratio between the number of metastatic lymph nodes and the LNH examined in the surgical specimen and patients were stratified into six subgroups according with the criteria used by Lee and coauthors: LNR0, LNR1 (0.01–0.05), LNR2 (0.06–0.1), LNR3 (0.11–0.2), LNR4 (0.21–0.3), and LNR5 (>0.3) [14].

Since current international guidelines recommend an evaluation of at least 15 lymph nodes for an appropriate staging of the disease [5], we furthermore categorized patients into those presenting with ≥15 nodes in the surgical specimen and those presenting with a LNH < 15.

### 2.5. HER2 IHC

Briefly, 4 *μ*m sections were deparaffinized in xylene and then placed in a graded series of ethanol. Sections were treated with citrate buffer (0.01 M sodium citrate (pH 6.0)) and heated in a microwave oven at 600 W (three times for 5 min each). Slices were then incubated with antibodies against HER2 (7269 M, 1 : 1000 dilution, Dako, Glostrup, Denmark) overnight at room temperature. Negative control sections were prepared by substituting the primary antibody with buffered saline. A semiquantitative approach was used for scoring the HER2 IHC reactivity, according to the criteria adopted by the ToGA study: HER2 0 (no reactivity or membranous reactivity in <10% of tumour cells), HER2 1+ (faint membranous reactivity in ≥10% of tumour cells); HER2 2+ (moderate complete, basolateral or lateral membranous reactivity in ≥10% of tumour cells); HER2 3+ (strong complete, basolateral or lateral membranous reactivity in ≥10% of tumour cells) [13].

### 2.6. Follow-Up

The follow-up of the patients has been conducted by telephone interviews with the following endpoints: overall survival (OS, all causes of death), disease free survival (DFS, first recurrence of the disease after the surgical treatment), and disease specific survival (DSS, death due to cancer of the stomach). The mean follow-up for the gastrectomy group has been of 41.0 months, whereas in the HER2 group has been of 49.4 months.

### 2.7. Statistical Analysis

Survival was analyzed using the Kaplan-Meier method by the log-rank test. All statistical evaluations including the ROC curve analysis were conducted with the statistical software MedCalc version 11.4.4.0.

## 3. Results

### 3.1. Patients

**Figure 1:**
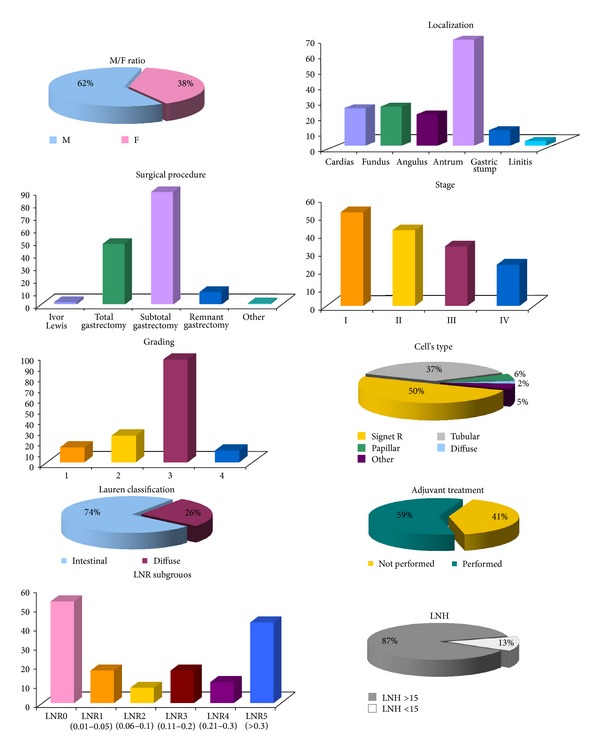
Clinical and pathological features of patients undergoing R0 gastrectomy for gastric adenocarcinoma 2003 to 2011 at our Department including the M/F ratio, the localization of the tumors and the surgical treatments, the stage of the diseases, the grading of the tumors, the LNR and the LNH subgroups, the cellular types and Lauren classification, along with the rate of adjuvant treatment performed in our series.

Figure 1 shows patient's clinical and pathological features of the 150 R0 patients undergoing gastrectomy for gastric adenocarcinoma at our Department from 2003 to 2011 (mean age 66; median 67; SD 11.8; range 31–88 years).

We reported a prevalence of males (62% versus 38%; M/F ratio 1.63), of antrum/pylorus tumors (45.3%), and thus of subtotal gastrectomies (59.3%). The average of lymph nodes harvested was of 27.78 lymph nodes (median: 26; SD 12.8). Overall the mean LNR was of 0.22 (median: 0.08; SD 0.28). Notably, the vast majority of our patients (87%) presented a LNH > 15, in accordance with the current international guidelines [5].

### 3.2. Survivals and Stage

**Figure 2:**
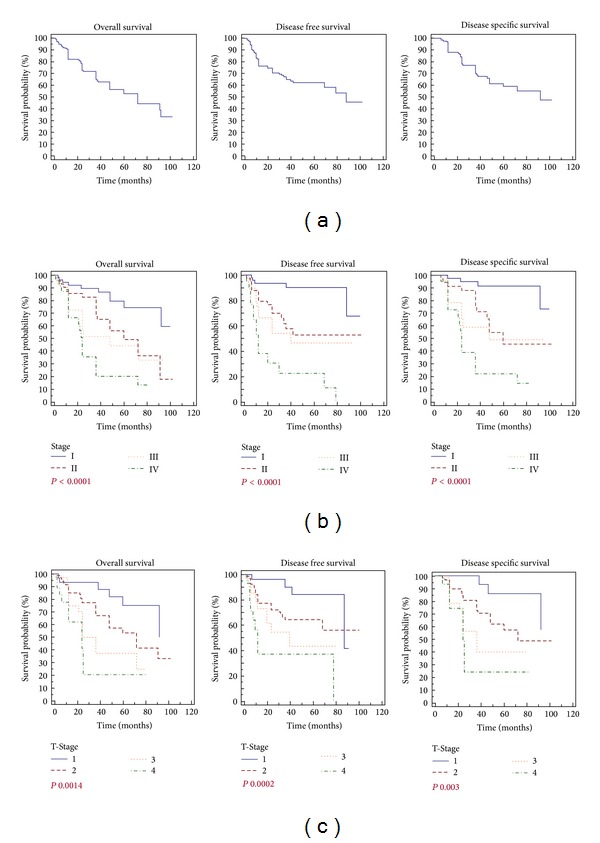
Gastric cancer and survivals. (a) Overall survival, disease free survival, and disease specific survival of R0 gastric resection for adenocarcinoma of the stomach; (b) overall survival, disease free survival, and disease specific survival analysis according to the Stage of the disease; (c) overall survival, disease free survival and disease specific survival according to the depth of tumors infiltration (T-Stage).

Figure 2 shows the survival analysis. As expected we documented a correlation between the stage of disease and the overall, disease free, and disease specific survivals (Figure 2(b), log rank test, *P* < 0.0001). Moreover we documented a correlation of statistical value between the depth of the tumor infiltration (T-Stage) and the prognosis of patients, including the OS (*P* 0.0014), DFS (*P* 0.0002) and DSS (*P* 0.003); see Figure 2(c).

### 3.3. Survivals and LNR-LNH

**Figure 3:**
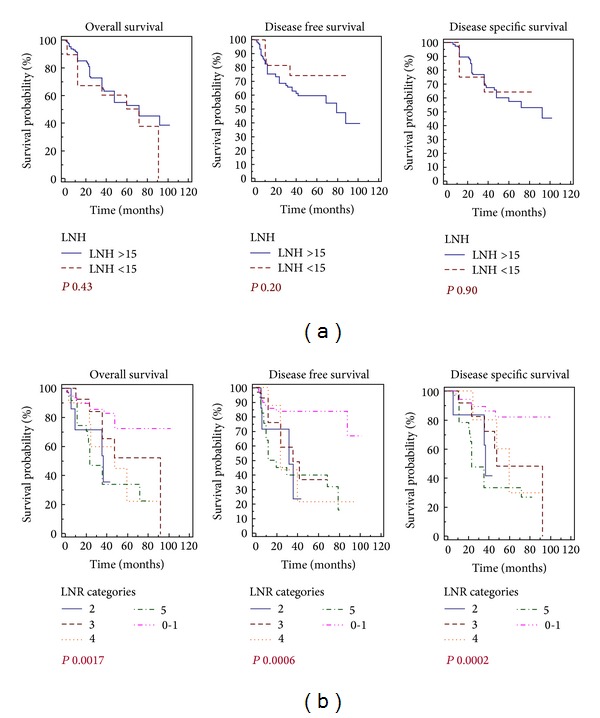
Gastric cancer and LNR/LNH related survivals. (a) Overall survival, disease free survival, and disease specific survival of R0 gastric resection for adenocarcinoma of the stomach according with the LNH subgroups; (b) overall survival, disease free survival, and disease specific survival analysis according to the LNR subgroups.

As highlighted in Figure 3, we reported a significant correlation between the LNR subgroups and the overall, disease-free, and cancer-specific survivals (log rank test respectively *P* 0.0017, *P* 0.0006 and *P* 0.0002, Figure 3(b)). Conversely the we could not detect a correlation of statistical value between a LNH > 15 nodes sampled in the surgical specimen and a better outcome of the patients (OS, DFS and DSS *P* ns; Figure 3(a)).

**Figure 4:**
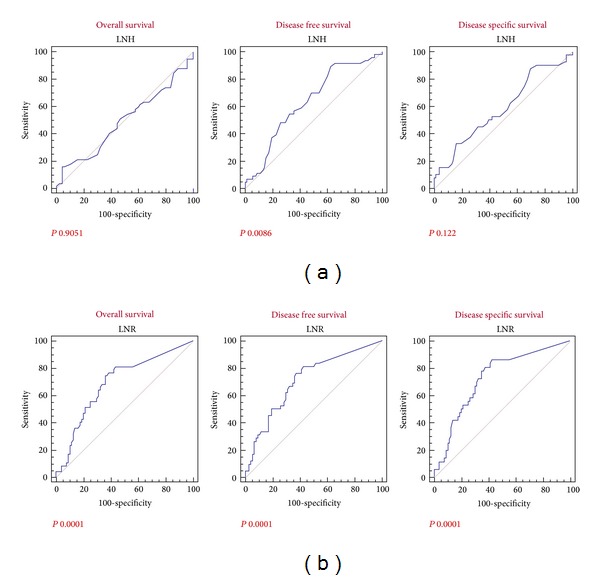
Gastric cancer and ROC curve analysis. (a) Overall survival, disease free survival, and disease specific survival ROC curves according to the LNH parameter; (b) overall survival, disease free survival, and disease specific survival ROC curves according to the LNR parameter.

We therefore performed a ROC curve analysis of the LNR and LNH values in relation to the overall, disease free and cancer-specific survivals in order to measure the performance of these factors analyzing the area under the curve (AUC). With respect to the LRN evaluation, the ROC curves displayed always left to the diagonal and the AUC analysis reported significant values for the OS, DFS and DSS (*P* 0.0001; Figure 4(b)). Conversely the evaluation of the ROC curves in relation to the LNH values documented a significant performance of this stratification exclusively for the DFS endpoint (*P* 0.0086; see Figure 4(a)).

### 3.4. Survivals and HER2 Overexpression

77 patients (mean age 62.8; median 64.0; SD 10.2; range 31–80 years) were included in this study. Patients were reported to be prevalently males (M/F 3.05); also the majority of these patients presented with an antrum localization (42.9%).  Table 1 reports the clinical and pathological data of this subgroup of patients. As highlighted in Table 1, 11.1% of the cancers were documented with a positive HER2 3+ IHC staining, whereas the 8.3% were documented with an equivocal staining (HER2 2+), the remaining being assessed as negative (HER2 0 62.5%, HER2 1+ 18.1%) [13].

**Figure 5:**
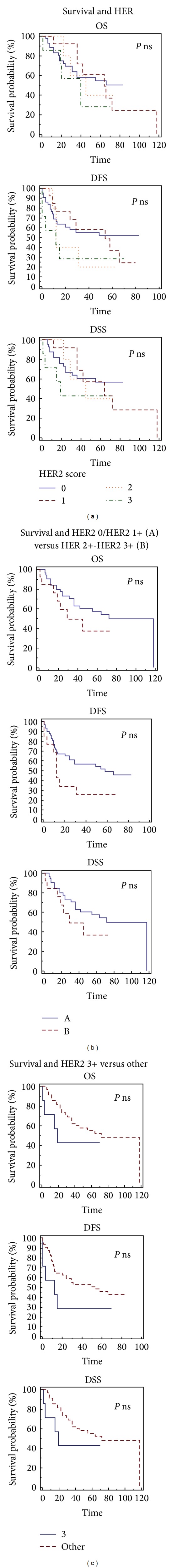
Gastric cancer and HER2 overexpression related survivals. (a) Overall survival, disease free survival and disease specific survival of gastric cancers according to the IHC scoring; (b) overall survival, disease free survival and disease specific survival analysis: negative tumors (HER2 0 and HER2 1+) versus equivocal/positive tumors (HER2 2+ and HER2 3+); (c) overall survival, disease free survival and disease specific survival: positive tumors (HER2 3+) versus other negative/equivocal tumors (HER2 0, HER2 1+, HER 2 2+).

Figure 5 reports the results of the correlation between HER2 overexpression and survival: even though none of the investigations reached a significance of statistical value, we reported a trend of better survivals for patients presenting with a negative or faint HER2 overexpression comparing with those scoring HER2 2+/HER2 3+ (*P* ns; Figure 5(b)); on the same extent a better outcome (not reaching a statistical value) has been documented comparing HER2 0/HER2 1+/HER2 2+ versus HER2 3+ patients (*P* ns; Figure 5(c)).

## 4. Discussion

Despite efforts in the detection along with the improvements of cure's strategies, gastric adenocarcinoma remains one of the major causes of cancer-related mortality.

Indeed in the MAGIC trial recently conducted in UK, the 3-5-year survival was of 36% in patients with operable disease who were assigned to peri-operative chemotherapy; nevertheless the 5-year survival rate for advanced or metastatic disease is around 5–20%, with median overall survival being less than 1 year [15–18].

The aim of the present study was to evaluate several clinical/pathological and molecular features as a possible prognostic factor of cancer-related survival in a consecutive series of patients who have undergone curative R0 gastrectomy and/or evaluated for the HER2 receptor overexpression.

The main translational objectives of our study and of similar studies conducted in this field would be to better profile the prognosis of gastric cancer patients and to select markers that could implement the current staging systems and possibly select patients candidate for intensive follow-up or for a tailored molecular treatment.

In this field, the molecular agent Trastuzumab (a monoclonal antibody that targets HER2) induces antibody-dependent cellular cytotoxicity, and inhibits the HER2-receptor signalling and cleavage [19]. Currently it is the standard of care for early and metastatic HER2+ breast cancer patients [20–22].

The recent ToGA study clearly demonstrated that Trastuzumab in combination with chemotherapy could improve survival of locally advanced/metastatic gastric/gastrooesophageal cancers showing overexpression of the HER2 receptor (HER2 3+ or HER2 2+ if FISH positive) [13].

Notably, even though a FISH amplification is routinely conducted in our centre for HER2 2+ tumors and Stage IV HER2 3+ or HER2 2+/FISH positive patients undergoing Trastuzumab treatment, we aimed this investigation to the evaluation of the HER2 overexpression by IHC *per se*, not considering the FISH evaluations or the possibly molecular therapies. Indeed, in our series HER2 2+ patients were the 8.3%; thus the evaluation of the FISH amplification within this category could be of limited value.

According to our results, we documented a trend of better outcome for patients presenting with a negative IHC (HER2 0, HER2 1+) versus equivocal/positive staining (HER2 2+, HER2 3+), even though not reaching a statistical value; the same has been reported pooling together negative with equivocal IHC versus positive tumors. These results confirm a previous investigation that we conducted in this field in a smaller series of patients [13].

On the other hand, we aimed this investigation to the implementation of the current nodal staging system with the LRN. Indeed, the LNR has been reported as an independent prognostic factor of overall survival in a large series of patients undergone who have R0 gastrectomy, regardless if more or less than 15 lymph-nodes were examined in the specimen [10]. This trend has been confirmed in each category of investigation (OS, DFS, and DSS), highlighting the effectiveness of this parameter as prognostic factor influencing the overall and the disease specific survival of patients.

Moreover the Italian Research Group for Gastric Cancer (IRGGC) investigated the prognostic value of the LNR in more than 1800 patients, documenting that LNR was an independent prognostic factor of OS, in agreement with our findings; authors moreover reported that the LNR led to the identification of subgroups of patients correlating with prognosis more homogeneously than the TNM classification system. [9].

Conversely another study recently reported that the number of metastatic nodes showed a greater accuracy than LNR in predicting the survival in a series of 96 patients [23].

Interestingly, we documented that the LRN performed better comparing to LNH as a prognostic factor, as outlined by the stratification of survivals by the Kaplan-Meier method and the ROC curves analysis.

A possible limitation of the present study might be the relative small number of patients analyzed that limited our analysis to the main outcome measures of translational impact (e.g. we did not include in our investigation the stratification of patients according to the tumor's cellular type).

However, it is important to highlight that we did not limit our analysis to the overall survival, but also included the disease free and cancer specific survival in order to better understand the prognostic value of this clinical/pathological and molecular features.

In conclusion and according to our analysis, it is worth correlating the clinical/pathological and molecular features of gastric cancer patients with survival since it might help in selecting markers that could implement the current staging systems and possibly in selecting patients with a higher risk of recurrence candidate for intensive follow-up (as the LNR) or for a tailored molecular treatment (as the HER2 overexpression IHC evaluation).

## Figures and Tables

**Table 1:** Clinical and pathological features of patients who have undergone IHC evaluation for HER2 over-expression in gastric adenocarcinoma from 2003 to 2011.

	*n *	%
Sex		
F	19	24.7
M	58	75.3
Age (years)		
Mean		62.8
Median; SD		64.0; 10.2
Range		31–80
Localization		
Cardias	16	20.8
Fundus	19	24.7
Antrum	33	42.9
Angulus	4	5.2
Anastomosis	1	1.3
Linitis	4	5.2
HER2 IHC score		
HER2 0	45	62.5
HER2 1+	13	18.1
HER2 2+	6	8.3
HER2 3+	8	11.1
Stage		
I	18	24.3
II	14	18.9
III	21	28.4
IV	21	28.4
Follow-up (months)		
Mean		49.4
Median; SD		59.0; 26.7
Range		3–83
